# Resistant tuberculosis in Maranhão, Brazil: a case series

**DOI:** 10.1186/s13104-016-2063-x

**Published:** 2016-05-04

**Authors:** Kenia Regina Oliveira Maia, Graça Maria de Castro Viana, Marcos Antonio Custódio Neto da Silva, Maria do Desterro Soares Brandão Nascimento, Victor Lima de Souza, Silvio Gomes Monteiro

**Affiliations:** Graduate Program in Parasitic Biology, University CEUMA, São Luís, MA Brazil; Nucleus of Basic and Applied Immunology, Department of Pathology, Federal University of Maranhão, São Luís, MA Brazil; Medicine School, Federal University of Maranhão, Scientific Scholarship of CNPq, São Luís, MA Brazil; Graduate Program in Adult and Child Health, Nucleus of Basic and Applied Immunology, Department of Pathology, Federal University of Maranhão, São Luís, MA Brazil; Medicine School, Federal University of Maranhão, São Luís, MA Brazil

**Keywords:** Tuberculosis, Anti-tuberculosis drugs, Resistance

## Abstract

**Background:**

*Mycobacterium tuberculosis* multidrug resistance, especially against rifampicin and isoniazid, places pulmonary tuberculosis in the list of emerging diseases. The misuse of therapeutic regimens is one of its main predisposing factors.

**Case presentation:**

Four clinical cases (three were brown and one black) with multidrug-resistant tuberculosis, treated in a reference hospital in the state of Maranhão, Brazil, were reported to evaluate the importance of radiological framework on disease evolution.

**Conclusion:**

The clinical framework showed a bad evolution and drug resistance, while radiology showed lung lesions, ranging from exudative infiltrates to lung parenchyma disintegration.

## Background

The adverse effects of anti-tuberculosis drugs are related to the high incidence of noncompliance to treatment [1]. Bacillus’ resistance to anti-tuberculosis drugs is related to wild strain mutations and the mutant selection process during treatment [2–4] in cases related to discontinuation or irregular treatment with standard regimens. Cases that led to primary resistance or acquired resistance, without contact, have also been found upon using specific drugs [5]. Cavitary tuberculosis cause outbreaks with higher resistance and mutant frequency [2].

This paper shows four clinical cases of patients with multidrug-resistant tuberculosis (MDR-TB) assisted in a reference hospital in the state of Maranhão, Brazil. In addition, the radiological framework importance on disease evolution was assessed.

## Case presentation

This paper consists of a retrospective study with 41 confirmed MDR-TB cases in 2003–2010. Only four cases are described in this series, as they had a complete supporting documentation.

### Case 1

A 33-year-old man, brown, married, was admitted in October 2006, with a history of Scheme III failure (indicated for therapeutic failure; 2 months with streptomycin, ethionamide, ethambutol and pyrazinamide, and 4 months with ethionamide and ethambutol) and resistance to rifampicin, isoniazid and streptomycin; negative HIV test; chest radiograph showed a right hemithorax fissure rectification and an exudative bronchiectasis lesion in the hemithorax. One year and 1 month later, fibro-atelectatic lesions were observed in the right upper lobe. The patient initiated the MDR-TB treatment 3 years later, having a positive sputum smear for acid-fast bacilli (AFB), confirmed by culture, with an oscillating pattern, and two positive cultures for *Mycobacterium tuberculosis*, with resistance to isoniazid, rifampicin, streptomycin and ethambutol. MR scheme for MR-X was conducted with ciprofloxacin, clarithromycin and pyrazinamide. Terizidone and bacilloscopies continued with an oscillating pattern. During 2 years and 6 months, two negative cultures were performed, with clinical resolution and radiological pattern of healing (Table 1).

### Case 2

A 27-year-old woman, brown, single, student, with a history of irregular treatment with regimen I in 2005 (indicated for new pulmonary and extra-pulmonary tuberculosis cases, except tuberculous meningitis; 2 months with rifampicin, isoniazid and ethambutol, and 4 months with rifampicin and isoniazid). HIV was negative. After 6 months, the patient developed productive cough, fatigue, fever episodes and gastrointestinal symptoms, then resumed with the tuberculostatic medication. The radiological framework showed residual pleural injury. Three years later, a cavitary lesion in the left upper lobe and bilaterally diffuse alveolar infiltrates were found when the enhanced scheme I was repeated, as having a positive sputum smear for AFB. A month later, bilateral lesions were found with cavities in the right upper lobe, positive smear and culture, and then the MDR-TB treatment began. Three months later, the positive culture remained, and a bilateral parenchymal fibrosis to the right was observed. A month later, the radiological examination showed bilaterally diffuse interstitial-alveolar infiltrate. Two months later, the radiological examination showed alveolar lesions in the apices, an atelectasis band to the right and a positive culture. Four years later, decreased lung size, atelectasis bands and cavitary lesions in the upper lobes were found. Three months later, bilateral bronchiectasis areas were observed. After three months, the patient was in a severe general condition. Five years later, a positive smear was found and an alternative scheme began. A month later, bilateral cavitation areas were observed, with right lung reduction. Three months later, radiological pattern persisted and positive smear with sensitivity tests indicating isoniazid and rifampicin resistance were found. Although the patient was treated, the severity evolved without improvement and the patient died 2 months later (Table 1).

### Case 3

A 38-year-old woman, black, homemaker. Chest radiography showed diffuse interstitial-alveolar infiltrates with pulmonary parenchyma, bubbles and bronchiectasis in the upper lobes. The treatment with scheme I began in 2006. It was abandoned, and the patient resumed treatment 1 year later with an enhanced scheme I. After a few months, the patient stopped taking medication, and the treatment resumed with the MDR-TB regimen. Three months later, being the smear positive, a positive culture for *M. tuberculosis* was observed in the susceptibility test, showing resistance to isoniazid and rifampicin. Six months later, *Mycobacterium hodleri* was identified by PCR. Seven months later, a radiograph showed large pneumatoceles in the upper lobes, left apical bronchiectasis, and fibro-exudative and cavitary lesions in the upper segments of lower lobes. Three months later, the patient was discharged after the cure of MDR-TB, returning repeatedly for 10 months, when the AFB smear was positive. Chest radiograph showed bubbles, bronchiectasis and fibrous residual lesions in the upper lobes (Table 1).

### Case 4

An 18-year-old woman, brown, single, peasant. She started the regimen I treatment in 2003, and abandoned it. One year later, the treatment was resumed with the enhanced scheme I, showing therapeutic failure. One year later, the MDR-TB treatment began. Two years later, the patient was treated for MDR-X tuberculosis. Bacilloscopy oscillated during the course of treatment. The culture trials conducted during treatment were positive for *M. tuberculosis*, showing a resistance to isoniazid, rifampicin and ethambutol. Six months later, a chest radiograph showed alveolar infiltrates in the left lung base. A year and 10 months later, the radiograph showed left lung parenchymal destruction, with cavitary lesions in the right upper lobe (Table 1).

**Table 1:** Clinical characteristics of patients with documented multidrug-resistant tuberculosis (MDR-TB)

Patient	Age	Tuberculosis duration, years	Drugs to which the patient was resistant^a^	Severe CXR findings^b^	Time to smear conversion, months	Adverse reactions to the treatment
1	33	3	INH, Rif, Eth, Stm	No	12	None
2	27	5	INH, Rif	Yes	–	GI
3	38	4	INH, Rif	Yes	–	None
4	18	7	INH, Rif, Eth	Yes	–	None

*CXR* chest radiograph, *Eth* ethambutol, *INH* isoniazid, *Rif* rifampin, *Stm* streptomycin, *GI* gastrointestinal

^a^Patients were tested for resistance to Eth, INH, Rif, PZA, Stm, Km, Cm, and Tha, as well as resistance to ciprofloxacin and cycloserine

^b^Cavitary and bilateral lesions

## Discussion

Although the literature describes a high toxicity of drugs used in the MDR-TB treatment [6], adverse effects were only observed in one case (25 %).

Through the report of four cases, the presence of severe pulmonary lesions with irreversible characteristics was radiographically evident, usually beginning with exudative lesions and parenchymal lung destruction. Case 3 shows a concomitant infection by *M. hodleri*, with very exuberant lung lesions. All patients described abandoned treatment, and had cavitary and fibro residual lesions and treatment failure, and showed no HIV coinfection, which is an association commonly described in the literature [7].

Chest radiography predicts tuberculosis prognosis by evaluating pulmonary lesions [7], and it is still the most used imaging method [8]. Cavitary lesions and residual fibrosis found related to multidrug resistance corroborate the literature [2].

In this study, 7.3 % of MDR-TB and 2.4 % of XDR were found, values lower than those reported for the USA. Marks et al. [9] found a prevalence of 36 % of MDR-TB and 56 % of XDR-TB in 2005–2007 [9]. Regarding drugs, resistance to rifampicin and isoniazid, as well as age (young adults), supported the findings of a study conducted in Congo [10].

In summary, MDR-TB is a major public health problem in Brazil, especially in poor areas such as the Brazilian North and Northeast.

## Abbreviations

AFB: acid-fast bacilli
HIV: human immunodeficiency virus
MDR-TB: multidrug-resistant tuberculosis
XDR: extensively drug-resistant tuberculosis
